# Context- and Template-Based Compression for Efficient Management of Data Models in Resource-Constrained Systems

**DOI:** 10.3390/s17081755

**Published:** 2017-08-01

**Authors:** Jorge Berzosa Macho, Luis Gardeazabal Montón, Roberto Cortiñas Rodriguez

**Affiliations:** 1 Electronics and Communications Unit, IK4-Tekniker, Calle Iñaki Goenaga 5, 20600 Eibar, Spain; 2 Computer Science Faculty, University of the Basque Country UPV/EHU, Paseo M. Lardizábal 1, 20018 Donostia-San Sebastián, Spain; pedrojoseluis.gardeazabal@ehu.eus (L.G.M.); roberto.cortinas@ehu.eus (R.C.R.)

**Keywords:** cyber physical systems, data models, compression, resource-constrained devices, ad hoc networks, Wireless Sensor Networks (WSN)

## Abstract

The Cyber Physical Systems (CPS) paradigm is based on the deployment of interconnected heterogeneous devices and systems, so interoperability is at the heart of any CPS architecture design. In this sense, the adoption of standard and generic data formats for data representation and communication, e.g., XML or JSON, effectively addresses the interoperability problem among heterogeneous systems. Nevertheless, the verbosity of those standard data formats usually demands system resources that might suppose an overload for the resource-constrained devices that are typically deployed in CPS. In this work we present Context- and Template-based Compression (CTC), a data compression approach targeted to resource-constrained devices, which allows reducing the resources needed to transmit, store and process data models. Additionally, we provide a benchmark evaluation and comparison with current implementations of the Efficient XML Interchange (EXI) processor, which is promoted by the World Wide Web Consortium (W3C), and it is the most prominent XML compression mechanism nowadays. Interestingly, the results from the evaluation show that CTC outperforms EXI implementations in terms of memory usage and speed, keeping similar compression rates. As a conclusion, CTC is shown to be a good candidate for managing standard data model representation formats in CPS composed of resource-constrained devices.

## 1. Introduction

The trend to integrate heterogeneous systems and devices into Cyber Physical Systems (CPS) demands interoperable communications and data models. Nevertheless, since many systems are composed of resource-constrained devices, a big effort is being made to provide those systems with protocols and tools adapted to their limitations. A general approach is to tackle the challenge at different layers. For example, we can find IEEE 802.15.4 [1] for the media access control layer, IPv6 over Low power Wireless Personal Area Networks (6LoWPAN) [2] in the case of the network layer and the Constrained Application Protocol (CoAP) [3] at the application layer.

A more current trend is the Web of Things (WoT) [4,5], which takes the Internet of Things (IoT) paradigm one step forward. WoT consists of supporting web standards directly on embedded devices by reusing and adapting standard web protocols to the constrains of such systems. WoT is totally based on application layer protocols, effectively abstracting lower layers, e.g., physical or transport layers, which are the ones showing the highest degree of heterogeneity and the main source of clashes in traditional IoT networks. In short, WoT promotes generic/standard interfaces in order to enhance overall interoperability and build loosely coupled services by providing mechanisms for highly configurable services/interactions. Apart from enhancing overall interoperability, web services enable the application of high level services, such as the “self-*” services family (where “self-*” stands for self-discovery, self-configuring, etc.), directly on top of the devices. In general, the benefits of WoT for all CPS domains are clear, but they are specially important for consumer electronics-targeted domains (domotics, entertainment, etc.), where improved interoperability across services/vendors and increasing richness of interactions with the “smart” environment would enhance user experience.

The efficient management of standard data model representation formats would ease the native use of high level data models and protocols (such as web services) in resource-constrained devices. In the context of this paper, we consider “resource-constrained” as low memory (<256 KB Flash/ROM and ∼10 KB RAM), limited processing capability (<48 MHz, typically 8–16 MHz) and an average consumption of a few μA due to energy source limitations and autonomy requirements.

Thus, this work considers the representation of data, which can be done using many different formats. We will focus on standard data model representation formats and, more precisely, W3C’s XML (Extensible Markup Language) as a main reference, even though other options such as JSON (JavaScript Object Notation) could also be considered. XML is widely extended as a data structuring format and is the basis for many application layer protocols, web services and related protocols, e.g., Simple Object Access Protocol (SOAP) [6] or Extensible Messaging and Presence Protocol (XMPP) [7].

XML has been designed to be human readable, with tokens codified as strings. This makes XML documents too large and too CPU demanding to be efficiently managed by resource-constrained devices. XML parsers need to deal with large amounts of string data and verbose documents, involving too much processing for energy- and processor-constrained devices. The size of XML documents puts some constrains on the required storing memory and transmission bandwidth. Resource-constrained devices usually have small memories of tens of KBytes, which should be able to store the XML document(s), as well as the application, operating system, communication libraries, etc. Additionally, the size of the XML documents has a direct impact on the number of message packets needed to transmit the whole document, which, as a consequence, affects the energy consumed by the device that communicates and, in the case of multi-hop networks, also by routing devices. For a survey on protocols targeted to resource-constrained devices, their characterization and limitations, readers are encouraged to consult [8].

In this work, we propose an approach based on templates, namely Context- and Template-based Compression (CTC). Roughly speaking, templates are extracted from the managed data model schema documents so that their representation can be replaced in the data model instance documents with a minimum number of references. Documents are then compressed (by using lossless-compression) following an algorithm that takes into account the context(s) of the data model’s schema.

The purpose of CTC is to reduce the resources needed to transmit, store and process data models compared to using the standard data representation formats (such as XML or JSON). First, by compressing the data model document instance, the quantity of messages needed to transmit the whole document is effectively reduced. Second, the use of templates minimizes the memory needed to store the data models’ schemas and the structures of the instances. Finally, the data model is codified in a more efficient format, resulting in a reduction of the required processing time. Tests have been performed in real hardware in order to evaluate our proposal. Results show that CTC outperforms other solutions and is a valid candidate for data model management in resource-constrained devices and networks.

In this paper, we also outline the communication model followed by CTC. Basically, templates are registered at execution time and are made available to the rest of the system. In order to manage data model schemas, nodes go through a preliminary discovery phase, where required templates and identifiers are downloaded from their storing location.

The paper is structured as follows: Section 2 presents the related work for this paper. Section 3 sets the concepts of CTC. Then, Section 4 introduces the compression and codification algorithm used in CTC. Section 5 describes the management and communication model used together with CTC. A performance study compared to Efficient XML Interchange (EXI) processor implementations is presented in Section 6. Finally, Section 7 summarizes and concludes the paper pointing out future steps.

## 2. Related Work

Although there are several XML compression algorithms, currently the most promising one seems to be EXI [9], adopted as a recommendation by W3C. For a comprehensible comparison of XML compression algorithms, readers are encouraged to consult [10]. EXI relies on a binary representation of XML, and it is designed to provide a considerable reduction on the size of the information in XML format (70–80%, as shown in [11]) and a high performance when encoding/decoding (6.7-times faster decoding and 2.4-times faster encoding according to [12]). In EXI, an XML document is represented by an EXI stream, which is composed of a header (containing encoding information) and a body (representing the data). Data are represented based on formal grammars to model redundancy. EXI uses a string table to assign “compact identifiers” to string tokens (such as qualified names and literals). Occurrences of string tokens found in the string table are represented using their associated compact identifier. The string table is dynamically expanded to include additional string values encountered in the document. When XML Schema information is available, the string table is initially pre-populated, allowing a much more efficient coding and compression.

However, EXI may be too complex to be efficiently implemented in resource-constrained devices. On the one hand, the implementation may require too much code memory or processing time. On the other hand, EXI requires the use of runtime memory allocation in order to accommodate schema deviations and grammar learning. The EXI Profile [13] recommendation proposes a series of configuration parameters and practices in order to reduce the memory needs of EXI implementations. EXI Profile is targeted to devices that are not allowed (either by design or convenience) to use arbitrary memory growth at runtime. The use of runtime memory is bounded by restricting the growth of string tables and the evolution of grammar(s), sacrificing some of the compression efficiency. However, these recommendations may not cover the resource limitations of the most resource-constrained devices. In contrast, CTC is specifically targeted to resource-constrained devices and makes use of templates and schema context information for energy-efficient management of standard data model representation formats.

The exploitation of XML templates is not a new concept. Extensible Style sheet Language Transformation (XSLT) [14] is the W3C standard for transforming XML documents into a different format, and its core is also based on template matching. An XSLT document is used to specify templates that match different portions of the original document. The XSLT document is in XML format, and templates are matched using XPath queries. When the XSLT processor finds a match, the code assigned to the template is executed, and the result is added to the output document. This turns XSLT into a very versatile and powerful tool. However, XSLT provides the template matching mechanisms, but does not specify any coding or compression algorithm, leaving this task to the user. XSLT also requires the parsing of two XML documents each time a transformation is performed: the XSLT document and the document/stream to be transformed. Thus, if XSLT were natively used in the device, it would have all of the drawbacks of natively parsing XML (the XSLT document) plus the overhead of parsing a second document/stream. In its last specifications, XSLT has been extended and also supports the transformation of non-XML documents through ad hoc parsing filters. In contrast, CTC defines templates as raw character strings, and it is not tied to any specific format.

Hoeller et al. [15,16,17] identified the inefficient management of XML formatted data in resource-constrained devices as a barrier to overcome in order to achieve full interoperability. They defined a series of mechanisms to efficiently manage XML in terms of processing, storing and transmission. They also provide a pre-compiler tool called $XOBE_{SN}$ [17]. This tool allows an easy integration of XML structures in C programs that are later translated into plain C, compilable with standard compilers. Additionally, it makes use of reused structures to efficiently store and process XML documents. The client/server communication model is based on XPath queries and optimized for this purpose. The use of pseudo XML structures in the code makes the solution proposed by Hoeller et al. heavily tied to XML. CTC uses a more natural data binding technique by using native C structures, giving a convenient abstraction of the underlying original data representation format. The work presented by Hoeller et al. does not provide a formal encoding or compression format for data transmission. The use of templates is suggested for the transmission of data, but few details are given.

## 3. Context- and Template-Based Compression Components

**Figure 1:**
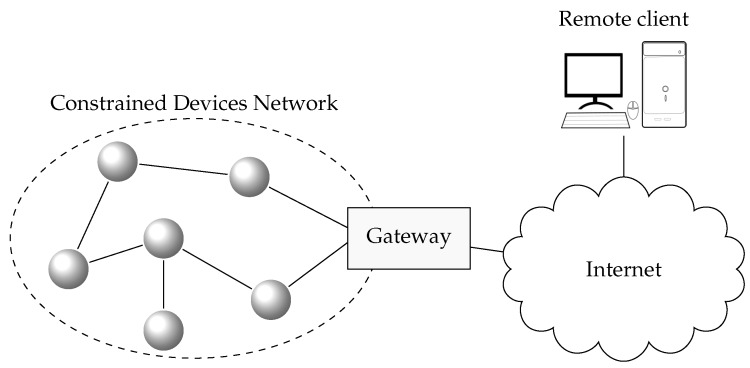
Basic architecture.

The main philosophy behind CTC is to use a data model representation encoding that is more efficient than standard formats, but that allows seamless transformation between the CTC format and the original format. CTC is conceived of as a part of a more complex distributed system. Figure 1 shows the simplified architecture of such a system, which is similar to communication architectures found in traditional Low Power Wireless Personal Area Networks (LPWPAN) and CPS in general: resource-constrained devices are deployed in a dedicated network, and an edge-router or gateway is used to access external networks (such as the Internet) and clients.

Devices interchange data with clients that either reside in the same local network or in external networks. Devices with constrained resources will be able to take advantage of CTC, while more powerful devices use the original format at the same time. On the one hand, when both the resource-constrained device and the client implement CTC, the communication will be end-to-end, with the gateway acting as a mere router. On the other hand, if the client does not implement CTC and makes use of the data models in their original format, the gateway will act as an application level gateway and translate the original format to CTC and vice versa. CTC allows for the transformation between the two formats to be done in a transparent way so as not to break interoperability.

Thus, CTC defines a data model structure representation that is able to describe the links between the items and templates that compose a data model. The proposed approach is intended to be generic and not tied to a specific data description format (such as XML or JSON). The data model’s specific schema is used to extract a generic graph that is independent of the schema’s original representation format, as well as the templates used to build the schema instances. We denote this graph a *Schema Context*.

A *Schema Context* contains all of the relevant schema information including individual nodes and links. This approach is similar to the W3C Document Object Model (DOM [18]), which is one of the most popular data models for representing, storing, accessing and processing XML documents. DOM represents XML documents as a tree-structure where everything is a node: the document itself, elements, attributes, etc. DOM also specifies a low-level Application Programming Interface (API) for accessing, processing and modifying XML documents. In a *Schema Context*, the data model schemas are also represented as graphs, and the same terminology is used to refer to the relationships between nodes (parent, child, sibling, etc.).

However, unlike DOM, a *Schema Context* only considers two types of nodes: *Element*s and *eContext*s (short form of “Element Context”). An *Element* node encapsulates the properties of an item of the original schema and its associated template. For instance, an *Element* contains the cardinality and whether it is a basic type (“string”, “integer”, etc.). An *eContext* node basically groups child *Element* nodes. Depending on its type, an *Element* node may have an *eContext*, which contains the list of child *Element* nodes. An *Element* with no *eContext* is a leaf of the *Schema Context* graph.

**Figure 2:**
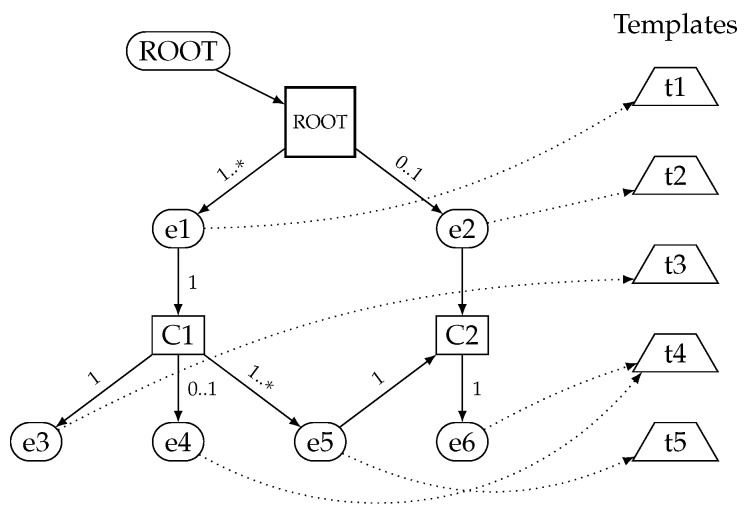
*Schema Context* graph example. Rounded nodes denote *Element*s, square nodes *eContext*s (short form of “Element Context”) and trapezium nodes templates. The numbers in the arrows indicate the cardinality: “1” one child, “1..*” one to many children, “0..1” none or one child (optional).

A simple *Schema Context* graph example is shown in Figure 2. The figure depicts the *eContext* and *Element* nodes, the links between them and associated templates. For instance, *Element* “e1” has an *eContext* “C1”, which in turn is the parent of child *Element*s “e3”, “e4” and “e5” with cardinalities “1”, “0..1” and “1..*”, respectively. Additionally, “e3”, “e4” and “e5” *Element*s are linked to templates “t3”, “t4” and “t5”, respectively. Note that *Element* “e4” shares its template (“t4”) with *Element* “e6”.

There are some other fundamental differences between DOM and *Schema Context*. DOM nodes only accept one parent (tree graph), while in the *Schema Context*, a node may have multiple parents. Although DOM is conceived of as a generic data model representation, it is especially targeted to XML and HTML formats, while the *Schema Context* does not make any specific assumption regarding the original format. Finally, DOM is used to represent any type of XML document, while *Schema Context* is only used to represent the data model schemas themselves, i.e., not the instances. Additionally, DOM representation of XML documents consumes much memory because the in-memory copy of a node keeps much information, and APIs tend to be heavy, producing verbose code. In contrast, the *Schema Context* is targeted to a minimum memory fingerprint, and the in-memory representation of a *Schema Context* only keeps the minimum information necessary to perform the codification.

**Figure 3:**
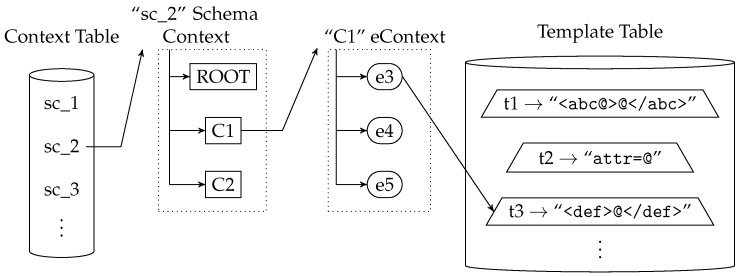
Example of the representation of Context- and Template-based Compression (CTC) components.

CTC itself has two main components. *Context Table*, which contains the *Schema Context*s, and *Template Table*, composed by the templates extracted from the schemas. Figure 3 shows a simplified representation of these two components. They are described with more detail in Section 3.1 and Section 3.2.

The encoding and decoding processes are executed following a specific algorithm, denoted *CTC Codification Algorithm*. In turn, the *CTC Codification Algorithm* uses the *Context Table* (or more specifically, the *Schema Context*s contained in the *Context Table*) as a reference in order to perform the encoding and decoding processes. The *CTC Codification Algorithm* is described in detail in Section 4.

### 3.1. Context Table

The *Context Table* contains all of the information of the data model schemas used by the device. Each entry of the *Context Table* is a *Schema Context* that contains the information related to the nodes in the schema, links between nodes, cardinality, links to templates and, in summary, all of the information needed to process a data model instance described by the schema.

A *Schema Context* is identified by the *URI* (Uniform Resource Identifier) and *SchemaId* attributes. The *URI* attribute must be unique, and it is used to globally identify the *Schema Context*. The *SchemaId* attribute is assigned at the device’s bootstrapping phase (as described later) and must be unique within the (sub-)network the *Schema Context* is used (for instance, within a wireless sensor local network).

A *Schema Context* is formally structured as a table where each entry is an *eContext*s node. In turn, each *eContext* entry contains a list with the child *Element* nodes. The first *eContext* of a *Schema Context* always belongs to the root *Element* node and indicates the entry point for the *CTC Codification Algorithm*.

Figure 3 shows a simplified representation of a *Context Table*, with the *Template Table* on the right side. The figure depicts the detail of a *Schema Context* with a *SchemaId* value of ‘2’ and *eContext*s “ROOT”, “C1” and “C2”. The figure also shows that *eContext* “C1” contains the child *Element*s “e3”, “e4” and “e5” and that *Element* “e3” is linked to template “t3”.

An *eContext* has the following attributes:

- *Id*: the unique identifier of the node, which is denoted by the *eContext*’s entry index within the *Schema Context*.
- *MultipleParents*: True if the *eContext* node is referenced by more than one *Element* nodes. False  otherwise.
- *Order*: the value of this attribute depends on the order the child nodes may appear in a data model instance document. If the order of the children is fixed and coincides with the order in which they are defined in the schema, the value is *fixed*. If the order is random, the value is *dynamic*. Finally, if only one single children can appear (among all of the ones defined in the schema for that particular node), the value is *choice*.
- *Children*: it contains the list of child *Element* nodes.

The *MultipleParents* attribute allows the reuse of the same *eContext* node by more than one *Element* node. The result is a better leverage of the memory as it avoids unnecessary duplicities. On the other hand, the *Order* attribute is used to perform the most efficient encoding of the children, tailored to the schema’s restrictions. This also avoids the parsing of irrelevant coded items, improving the overall processing speed. The most efficient encoding is provided by *fixed* children, followed by *choice* and, finally, *dynamic* as the worst case. The *choice* order value is a special case of *dynamic* order, where the order is also unspecified, but only one children can appear.

An *Element* node is composed of the following attributes:

- *Template*: a reference to the entry in the *Template Table* that contains the template for this *Element* node.
- *Type*: data type of the *Element* node. The data type can be either a basic type, a *constant*, a *complex* or a *schema*. For the basic data type case, the following types (inherited from the EXI [9] specification) are supported: *binary*, *boolean*, *decimal*, *float*, *integer*, *date-time* and *string*.
- *IsOptional*: True in case the cardinality of the child *Element* is 0..m, where m>0, and False  otherwise.
- *IsArray*: True in case the child *Element* can appear consecutively more than once, i.e., those children that have cardinality n..m, where m>n and n>1.
- *Context*: if the *Type* attribute is *complex*, *Context* attribute contains this child‘s *eContext*. In the case in which the *Type* attribute is *schema*, *Context* is equal to the *Schema Context* that describes the schema. A special value is used to represent a “<*any*>” schema, i.e., an unspecified schema. Basic types and *constant* type do not make use of the *Context* attribute.

The *Type* attribute is key in order to use the most efficient compression encoding for each data type. This also translates to average better processing performance as parsing/compressing with dedicated encoding is usually more efficient than processing plain string texts.

The *IsOptional* and *IsArray* attributes are used to codify the cardinality of the *Element* node. On the one hand, the *IsOptional* attribute identifies items that may not appear, removing the need to codify and process missing items. On the other hand, the *IsArray* attribute allows the codification of items’ repetitions by reusing the same *Element* (and its template) without the need for in-memory duplications. Additionally, a template can be referenced by more than one *Element*. In this way, *Template Table* entries are reused when possible, reducing memory requirements.

Table 1 shows the *Schema Context* table associated with the example data model *Schema Context* in Figure 2. For instance, as can be seen in Table 1, *Element* “e1” is linked to template “t1”, is of the *complex* type (thus, it has an *eContext*, “C1”) and is a non-optional array (cardinality “1..*”) with *IsOptional* to False and *IsArray* to True. As another example, *Element* “e4” is a leaf node (no *eContext*) of *string* basic type, and it is optional (cardinality 0..1) with *IsOptional* to True and *IsArray* to False. Additionally, *Element* “e4” is linked to template “t4” together with *Element* “e6”. Finally, note how *eContext* “C2” has *MultipleParents* attribute set to True and its linked by *Element*s “e2” and “e5”.

### 3.2. Template Table

**Figure 4:**
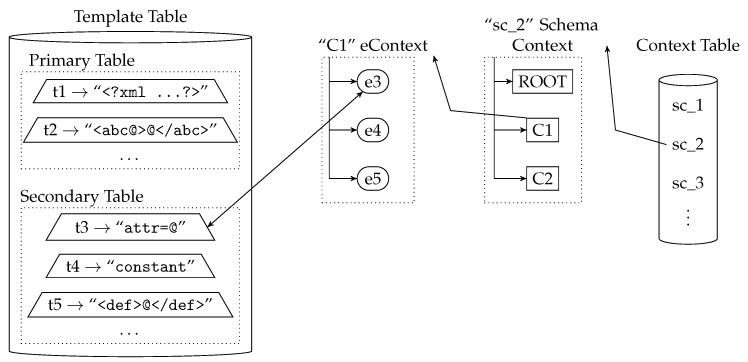
*Template Table* structure detail.

The *Template Table* stores the list of templates of the schemas used by the device. Basically, templates are represented by using a character string format. The *Template Table* also contains the position of the place-holders that represent the extension/nesting points for each template, i.e., where the templates of the child nodes or nested data models are inserted. Figure 4 shows the simplified *Template Table* structure with example content. In the figure, the place-holders of the example templates are represented with the character ‘@’.

The *Template Table* is structured and designed to provide efficient template searching and matching. As can be seen in Figure 4, the *Template Table* is divided in two sub-tables: *Primary Table* and *Secondary Table*. The *Primary Table* only contains the templates of the valid starting items of a data model, according to the structure described in the data model’s schema. That is, the schema defines which items must appear first in a valid instance document and only the templates of these items are included in the *Primary Table*. For instance, in the XML case, the *Primary Table* will include the templates of the XML global elements. The *Secondary Table* contains the templates of all of the remaining items.

In addition to the information related to the template representation, each table entry also contains information about the *Element* nodes that reference the template. This simplifies the matching between the original format, the templates, the *Element*s and their *eContext*s, thus improving and optimizing the searching, matching and codification processes.

However, templates are only needed when the data model has to be transformed from/to the original format. As will be explained later in Section 5.2, resource-constrained devices do not need to include the *Template Table*, reducing the memory needs. If the *Template Table* is needed in order to transform from/to the original format, two distinct cases are considered: decoding and encoding.

When a coded stream is decoded to retrieve the data model in the original format, the coded stream is parsed using the information in the *Context Table*, and templates are merged together as the items are processed. In this case, the *Template Table* acts as a mere container of templates.

In the other case, when a data model in the original format has to be codified to CTC, the *Template Table* assumes a more active role. First, the *Primary Table* is used as the entry point for the encoding process, and it is searched for a valid match. Once a match is found, the associated *Element*s and *eContext*s are recursively navigated until the full document is parsed. This searching strategy improves the search speed by reducing the searching range.

In summary, the *Template Table* has two main purposes. On the one hand, the *Template Table* is used during the decoding phase to rebuild the codified data model to its original format. On the other hand, it servers as a pattern matching reference during the codification process in order to search data model instances in their original format, match the template patterns and, finally, extract the associated *Element* and *eContext* nodes.

### 3.3. Schema Context and Template Table Creation

As explained in Section 3.1, the *Context Table* contains the list of all of the *Schema Context*s used by the device. Each *Schema Context* is created by processing the individual data model schemas. As the schema is processed, each *eContext* and *Element* is created strictly following the order in which the items are defined.

Basically, there are five pieces of information per item that need to be extracted from the data model schema in order to build the *Schema Context* and *Template Table*: the links between the items, the cardinality of those links, the order in which the children items can appear, the type of the item and, finally, the template that represents the structure of the item in the original format. How this information is gathered from the schema and processed is described in detail in the following paragraphs together with Algorithm 1. In order to avoid confusion, we will use the more generic term *node* to refer to a node of the original schema format (such as an “element” or “attribute” in XML or “property” in JSON) and the specific terms *Element* and *eContext* to refer to the respective nodes of the *Schema Context*.


**Algorithm 1:**
*Schema Context* creation 
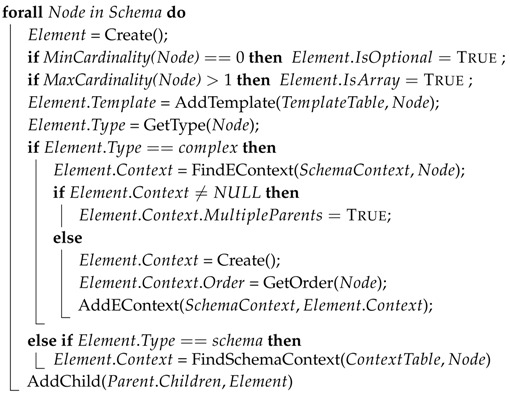



If the cardinality related to the node is 1, the *IsOptional* and *IsArray* attributes are set to False. If the node has cardinality n..m, where n=0, *IsOptional* is set to True. If m>1, then *IsArray* is also set to True. Then, the template is added to the *Template Table*, and the *Template* attribute is set to the assigned index within the *Template Table*.

Once cardinality attributes and templates have been processed, the node’s type is added to the *Type* attribute. In case the node’s type is *complex*, the algorithm checks whether its context already exists in the *Schema Context*. In case the context already exists, the *MultipleParents* attribute is set to True. On the contrary, if it does not exist, a new *eContext* is created, and the *MultipleParents* attribute is set to False.

In Case (a), the order of appearance of children is fixed, and in (b) the appearance matches the order defined in the schema; the *Order* attribute is set to *fixed*. If the appearance order of the children can vary dynamically, the *Order* attribute is set to *dynamic*. In case only one of the children can appear, the *Order* attribute is set to *choice*.

Finally, the *eContext* is added to the *Schema Context*.

If the node’s type is *schema*, the *Context* attribute is set to the associated *Schema Context*. For those cases where the nested schema is unknown a priori, the *Context* attribute is set to the special value “<*any*>”.

Finally, the new child *Element* is added to the *eContext* of the parent *Element*.

Once the schema has been processed and the *Schema Context* has been created, a process called *Context Collapsing* is performed. This process reduces the number of *eContext*s, *Element*s and templates without any loss of information. If (1) the *Order* attribute of an *Element*’s and a child’s *eContext*s are both *fixed*, (2) the child is neither optional nor array (i.e., IsOptional= False and IsArray= False) and (3) the child’s *eContext* only has one parent (i.e., MultipleParents= False), then the *eContext* and template of the child *Element* are merged together with the *eContext* and template of the parent *Element*.

*Context Collapsing* is executed starting from the root node in a recursive way, for each *eContext* and its child *Element*s. In practice, this process merges together nested fixed contexts, reducing the effective processing time, as fewer iterations and accesses to the *Context Table* and *Template Table* will be necessary.

### 3.4. From XML Schema to Schema Context

The previous section described the general algorithm and approach to create a *Schema Context* from a generic data model schema. Although the algorithm is generic, it has to be specifically implemented for each data format type, as the mapping of the schema to a *Schema Context* is data format specific. This section describes the specific case of the algorithm application to an XML Schema. Although a full detailed explication of the mapping of every single node type described in the XML Infoset is out of the scope of this paper, we give here an overview of the most relevant and representative use cases.

XML complex and simple elements are transformed into CTC *eContext*s. The value of *Order* attribute will vary depending on whether the containers XML order indicator is “all” (*dynamic*), “choice” (*choice*) or “sequence” (*fixed*).

XML element particles are mapped as *Element*s. The cardinality of the *Element* will depend on the XML occurrence indicators “maxOccurs” and “minOccurs”. XML attributes are mapped in a similar way as XML elements, but they are grouped into a single child *Element* with a dynamically ordered *eContext*.

An optional child *Element* containing the XML prolog is always added to the root *eContext*, followed by any relevant global definition (such as namespaces and prefixes). Global XML elements and attributes are also added as *Element*s to the root *eContext*.

Each XML namespace is transformed into a different *Schema Context*. If the XML type of an XML element or attribute belongs to a namespace other than the current one, the *Element Type* will be of *schema* type, and the *Context* will be assigned to the *Schema Context* of the relevant namespace. If the XML element or attribute is of “<*any*>” type, the *Element* *Type* will also be of the *schema* type, but the *Context* is specially marked to represent the special value “<*any*>”.

#### Context Table and Template Table Example

**Figure 5:**
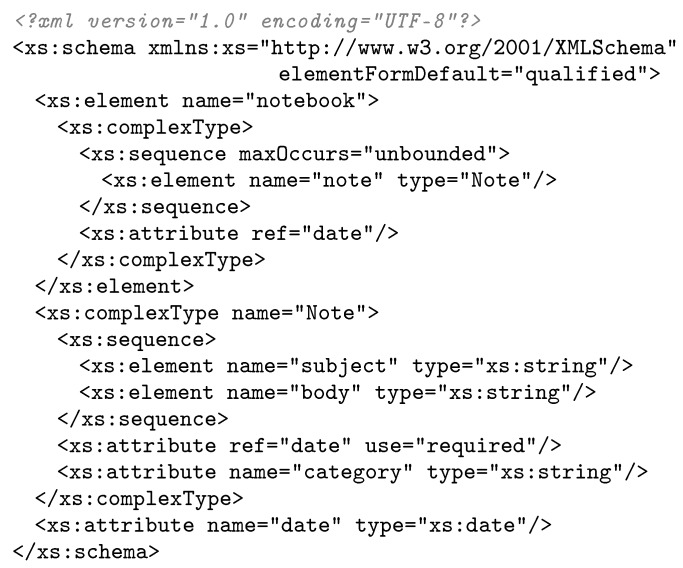
*Notebook* XML Schema document.

**Figure 6:**
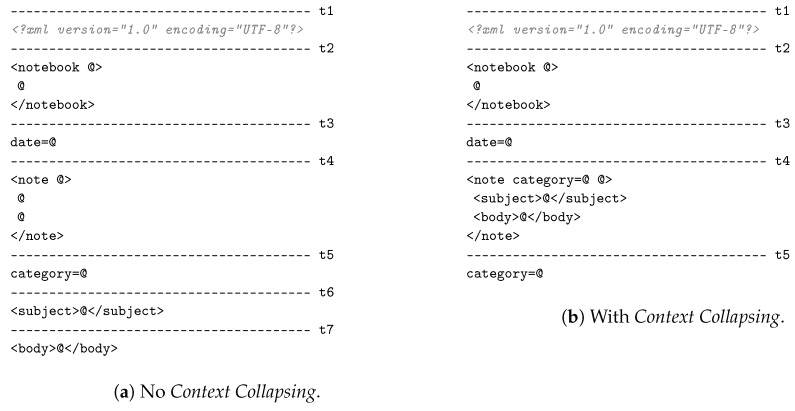
*Template Table Notebook* example. Symbol ‘@’ is used to represent the place-holders’ positions.

We present an example of an *Schema Context* and *Template Table* generated from an XML Schema. To this end, we use the *Notebook* XML document example proposed by Peintner et al. [19]. Figure 5 shows the original *Notebook* XML Schema example. Figure 6 shows the *Template Table* generated before performing *Context Collapsing* (see Figure 6a) as described in Section 3.3 and after *Context Collapsing* (see Figure 6b).

As can be appreciated in Figure 6, templates are merged together after *Context Collapsing* is performed, eliminating in the process the unneeded *eContext* and *Element* nodes. The result is a more compact *Schema Context* and *Template Table*. Finally, Table 2 shows the *Schema Context* generated after *Context Collapsing*. This table is related to the *Template Table* shown in Figure 6b and contains the *eContext*s and *Element*s after pruning the unneeded items.

### 3.5. Other Data Model Representation Formats

In this paper, XML has been used as the primary example to show the capabilities and mechanisms that form CTC. However, CTC can be used with any other data model representation format as long as the information regarding the structure of the data model can be extracted from a schema or by any other means. In a similar way as with the XML case, this information is used by CTC to build the *Context Table* and *Template Table* that will be used for the CTC compression and management processes.

As a simple additional example, we can consider another popular format such as JSON. In this case, the data model structure information needed by CTC can be extracted from a JSON Schema and mapped to a *Context Table* and *Template Table*. For instance, cardinality information can be inferred from the “required” and “array” JSON properties. The *Order* attribute of all of the *eContext*s would be “dynamic” because JSON does not enforce any order for the properties of a JSON object.

As the complexity of the format and related schema grows, so does the CTC schema mapping and encoding process. However, this complexity is mostly concentrated in the mapping of the schema to the *Context Table* and *Template Table*. Additionally, different techniques can be applied (such as the *Context Collapsing* method) to the table building in order to relieve the resource-constrained devices from the runtime overhead.

## 4. CTC Codification Algorithm

In this section, we describe the generic rules that, applied together, form the *CTC Codification Algorithm*, used to perform the encoding and decoding processes. The rules define the actions to perform for each node, based on the information available in the *Context Table*. The rules are grouped and formalized using a set of equations that represent the different steps involved in the encoding/decoding process of each node.

We define the following terms:

- We denote $e_{0}$…$e_{n-1}\in C$ as the ordered list of child *Element*s of the *eContext* *C* where *n* is the total number of *C*’s children.
- We denote $e_{0}^{\prime }$…$e_{m-1}^{\prime }\in C$ as the unordered list of child *Element*s of the *eContext* *C*, where *m*, m⩽n, is the number of *C*’s children actually appearing in the data model instance document.
- The Trim(x,y) function trims the representation of *x* to $\left\lceil \log_{2}y\right\rceil$ bits. Optionally, the form $x_{y}$ is also used to represent *x* with *y* bits. Thus, $Trim\left(x,y\right)=x_{y}$.
- The symbol ⊕ represents the concatenation of two bit arrays.
- The Pos(C,e) function returns the position index of the child *Element* *e* within *eContext* *C*. Note that, for an ordered child *e*, $Pos(C,e_{i})=i$ where 0⩽i<n. However, for an unordered child $e^{\prime }$, $Pos(C,e_{i}^{\prime })=i$ may not be True.

Four set of rules are defined together with the corresponding equations: $Cod_{S}\left(s\right)$ for *Schema Context*s, $Cod_{\mathrm{EC}}\left(C\right)$ for *eContext*s, $Cod_{E}\left(C,e\right)$ for child *Element*s and $Cod_{T}\left(e\right)$ for data types. $Cod_{S}\left(s\right)$ is always applied first.

Rule1: If the *SchemaId* of a (nested) schema is not known a priori (i.e., =<*any*>), the *SchemaId* must be codified before the root *eContext* of the schema is processed. Otherwise, the codification of the root *eContext* of the (nested) schema is processed directly.

(1) $$Cod_{S}\left(s\right)=\left\{\begin{array}{cc} Cod_{\mathrm{EC}}\left(s\right) & \;SchemaId(s)\neq <any>\\ \; & \;\\ SchemaId\left(s\right)⊕Cod_{\mathrm{EC}}\left(s\right) & \;SchemaId(s)=<any> \end{array}\right.$$

At the beginning of a coding/decoding process, Equation (1) is always used first. Thus, all CTC streams start with the *SchemaId* of the data model’s schema, followed by the root *eContext*.

Rule2.a: If the order of the child *Element*s is fixed, the codification of the *eContext* is equal to the concatenation of the children’s codification, following the same order the children are defined in the *Children* list attribute.

Rule2.b: If the order of the child *Element*s is independent of the order defined in the schema, a prefix equal to the child *Element*’s index plus one is added to the codification of each of the children. If not all of the children are present, a prefix of $0_{1}$ is used to indicate the end of the children list.

Rule2.c: If only one of the children can appear, a prefix equal to the child *Element*’s index is added to the codification of the children.

The following equation groups Rules 2.a, 2.b and 2.c.

(2) $$Cod_{\mathrm{EC}}\left(C\right)=\left\{\begin{array}{cc} Cod_{E}\left(C,e_{0}\right)⊕\ldots ⊕Cod_{E}\left(C,e_{n-1}\right) & \;Order(C)=fixed\\ \; & \;\\ \begin{array}{c} Trim\left(Pos\left(C,e_{0}^{\prime }\right)+1,n+1\right)⊕Cod_{E}\left(C,e_{0}^{\prime }\right)⊕\ldots \\ \ldots ⊕Trim\left(Pos\left(C,e_{i}^{\prime }\right)+1,n+1\right)⊕Cod_{E}\left(C,e_{i}^{\prime }\right)\\ ⊕Trim(0,n+1) \end{array} & \;Order(C)=dynamic\\ \; & \;\\ Trim\left(Pos\left(C,e_{i}^{\prime }\right),n\right)⊕Cod_{E}\left(C,e_{i}^{\prime }\right) & \;Order(C)=choice \end{array}\right.$$

$Cod_{\mathrm{EC}}$ is used to codify *eContext*s. As can be seen in Equation (2), the codification of an *eContext* depends mainly on the *Order* attribute. CTC defines a *strict* mode where the items of a schema are always codified strictly following the order defined in the schema. In this mode, all of the *eContext* nodes where condition Order=dynamic applies are considered to be *fixed*. The *strict* mode provides a more compact compression at the cost of some of the flexibility of CTC. However, this mode is ideal for resource-constrained devices, as it is straightforward for the device to codify the data models respecting the items’ definition order.

Rule3.a: If an *Element* is not an array, nor optional, the codification is equal to the codification of the *Element*’s type.

Rule3.b: if an *Element* is optional, but not an array, a $1_{1}$ prefix is added to the codification, followed by the *Element*’s type codification. In case the *Order* of the parent *eContext* is not *fixed*, the prefix is omitted. If the optional *Element* does not appear, a $0_{1}$ will be added to the codification.

Rule3.c: if an *Element* is an array, a $1_{1}$ prefix is added to each of the *Element* occurrences, and a $0_{1}$ is added when no more occurrences remain.

Equation (3) groups Rules 3.a, 3.b and 3.c.

(3) $$Cod_{E}\left(C,e\right)=\left\{\begin{array}{cc} 1_{1}⊕Cod_{T}\left(e\right) & \;\begin{array}{c} \left(IsOptional(e)=TRUE)\;\&\;(IsArray(e)=FALSE\right)\\ \&\;(e\neq null)\;\&\;Order(C)=fixed \end{array}\\ \; & \;\\ 0_{1} & \;(IsOptional(e)=TRUE)\;\&\;(e=null)\\ \; & \;\\ \begin{array}{c} 1_{1}⊕Cod_{T}\left(e\right)⊕\ldots \\ \ldots ⊕1_{1}⊕Cod_{T}\left(e\right)\\ ⊕0_{1} \end{array} & \;(IsArray(e)=TRUE)\;\&\;(e\neq null)\\ \; & \;\\ Cod_{T}\left(e\right) & \;otherwise \end{array}\right.$$

Finally, the following rules are used to process *Element*’s types:

Rule4.a: If the *Element* is a basic type, the built-in EXI data type representation is used to codify the *Element*’s value.

Rule4.b: When the *Element* is of *complex* type, the equation $Cod_{\mathrm{EC}}$ is used to codify the *Element*’s context.

Rule4.c: If the *Element* is of *schema* type, the equation $Cod_{S}$ is used to codify the *Element*’s context.

(4) $$Cod_{T}\left(e\right)=\left\{\begin{array}{cc} Cod_{\mathrm{EC}}\left(Context\left(e\right)\right) & \;Type(e)=complex\\ \; & \;\\ Cod_{S}\left(Context\left(e\right)\right) & \;Type(e)=schema\\ \; & \;\\ EXI\_basic\_type(e) & \;otherwise \end{array}\right.$$

### Codification Example

**Figure 7:**
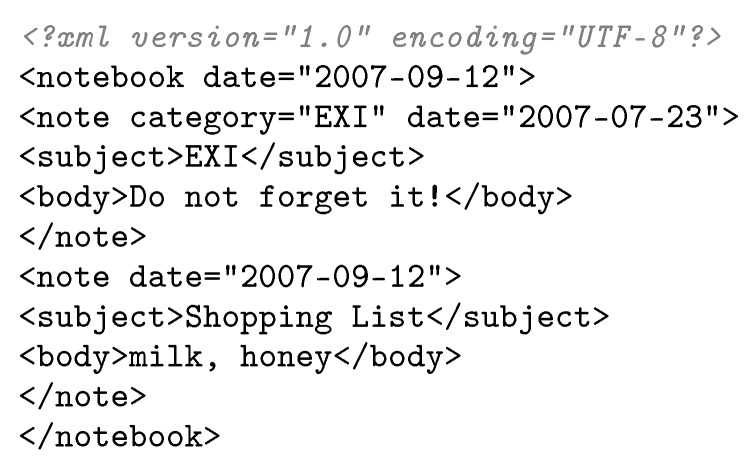
Schema example instance.

In order to clarify the application of the rules and equations explained in the previous section, the step by step codification of the XML instance shown in Figure 7 (which follows the *Notebook* schema of Figure 5) is described here. For simplicity, the example below only expands the first occurrence of the XML element “*note*”.

First, the *SchemaId* of the schema is codified, followed by the root *eContext*:

 CodS(sNOTEBOOK)⇒SchemaId(sNOTEBOOK)⊕CodEC(CROOT)        

Next, the *prolog Element* of the root *eContext* is processed, followed by the content of the data model instance:

 CodEC(CROOT)⇒CodE(CROOT,ePROLOG)⊕CodE(CROOT,eCONTENT)CodE(CROOT,eCONTENT)⇒CodT(eCONTENT)⇒CodEC(CCONTENT)        

The *notebook* XML element is codified into the stream taking into account that the *eContext Order* is *choice*:

 CodEC(CCONTENT)⇒01⊕CodE(CCONTENT,enotebook)CodE(CCONTENT,enotebook)⇒CodT(enotebook)⇒CodEC(Cnotebook)        

*notebook eContext* contains two *Elements*, one for the XML attributes and another for the *note* XML element:

 CodEC(Cnotebook)⇒CodE(Cnotebook,enotebook_att)⊕CodE(Cnotebook,enote)        

*note Element* is an array with length two:

 CodE(Cnotebook,enote)⇒11⊕CodT(enote)⊕11⊕CodT(enote)⊕01        

*note eContext* contains three *Elements*, one for the XML attributes and another two for the *subject* and *body* XML elements:

 CodT(enote)⇒CodEC(Cnote)⇒CodE(Cnote,enote_att)⊕CodE(Cnote,esubject)⊕CodE(Cnote,ebody)        

The *note_att eContext* contains the attributes of the *note* XML element. It is a *dynamic eContext* with two child *Elements*:

 CodE(Cnote,enote_att)⇒CodT(enote_att)⇒CodEC(Cnote_att)⇒⇒12⊕CodE(Cnote_att,edate)⊕22⊕CodE(Cnote_att,ecategory)        

Finally, basic type *Elements* are directly encoded using the EXI codification standard for built-in EXI data type representations. For instance, for the *subject Element* of type *string*, the value is codified as:

 CodE(Cnote,esubject)⇒CodT(esubject)⇒38⊕“EXI”        

## 5. Context Table Management and Communication Model

The functionalities provided by CTC are encapsulated in a library. These functionalities include the management of the *Context Table* and *Template Table*, as well as the execution of the *CTC Codification Algorithm*. This library is embedded and used by the resource-constrained devices in order to access the functionalities offered by CTC and to encode/decode data streams.

**Figure 8:**
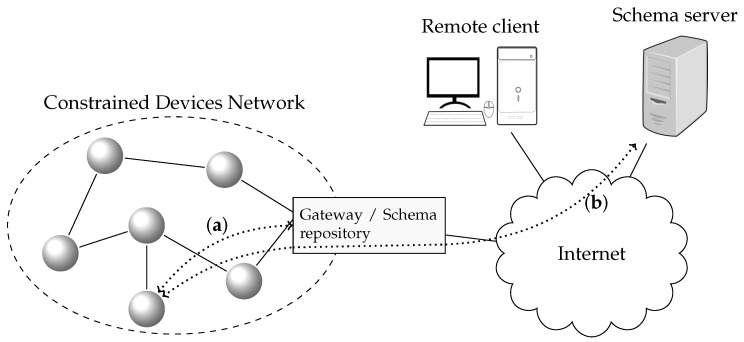
Template location. (**a**) at the node, (**b**) at an external server.

However, CTC alone does not provide all of the functionalities needed to be directly used in a CPS. CTC is conceived of as a component within a distributed system, such as the one shown in Figure 8: resource-constrained devices are deployed in a local network, and an edge-router or gateway is used to access external networks, such as the Internet.

In CTC, devices need to know the *Context Table*s and *Template Table*s (and their identifiers) associated with the data models they are using. This information is made available by the *Schema Repository*. Devices use the *Schema Repository* in an initial dissemination phase, in which *Template Table*s and *Context Table*s are distributed and registered in order to manage the schemas of the used data models. Although it is not required in all cases, for convenience, the *Schema Repository* is depicted at the gateway itself in Figure 8.

Depending on the application domain, resource-constrained devices interchange data with clients that may reside in the same local network or in external networks. If both the resource-constrained device and the client implement CTC, the communication will be end-to-end, with the gateway acting as a mere router. Note that the *Schema Repository* must be accessible from both the device and client, in order to satisfy the *Schema Context* dependencies during the bootstrapping phase.

In case the external clients do not implement CTC (i.e., they make use of the data models in their original format), the gateway will act as an application level gateway and translate the original format to CTC and vice versa. In this case, the gateway must contain a *CTC Library* in order to have access to CTC functionalities. However, the use of CTC is effectively hidden to the clients, and thus, the *Schema Repository* does not need to be externally accessible.

The following sections explain in more detail the schema registration process, as well as the use and implementation of the *CTC Library* in the resource-constrained devices and gateway.

### 5.1. Schema Registration

When a device joins the network for the first time, it can start a schema registration process. Schemas are registered in a centralized *Schema Repository*, usually located at the gateway. Devices use the URI of the data model schema to register. When the *Schema Repository* receives a registration request, it first checks whether that schema is already registered. In that case, the associated *SchemaId* is returned to the device. If the URI is not registered yet, the *Schema Repository* generates a new *schemaId*.

When registering a schema, an associated URL is provided so that its data model schema can be accessed and downloaded. Schemas can be stored in the device itself (see Figure 8a) or at an external server (Figure 8b). Once the *Schema Repository* has downloaded a schema, it generates the *Context Table* and *Template Table*. As an efficiency improvement, the *Schema Repository* could also pre-load a set of standard schemas or download already pre-compiled *Context Table*s.

Note that resource-constrained devices only need to store the schemas of the data models they actually use. Moreover, if the schemas are stored in an external server and are accessible by the clients, they can be totally stripped from the device.

Once the registration process is finished, the *schemaId*, *Context Table* and *Template Table* are available in the *Schema Repository* and will be accessible from local and remote clients during the bootstrapping phase.

The registration process does not assume any underlying protocol. For instance, a straightforward approach could be implemented by using CoAP [3] and GET/POST actions to gather/register the schemas together with CoRE Link Format [20] for discovery purposes.

### 5.2. CTC Library

**Figure 9:**
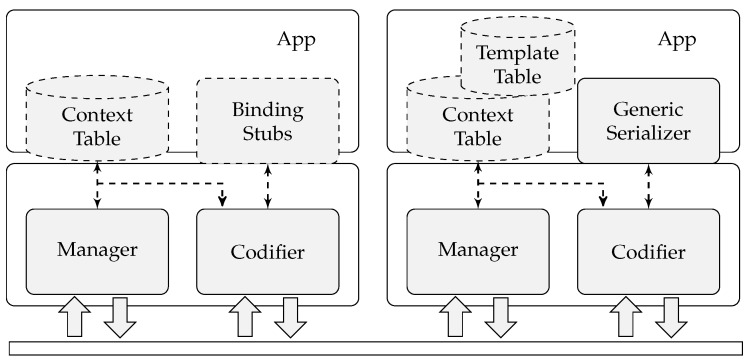
Architecture of the *CTC Library*. Dotted components are generated by the *CTC Compiler*.

As can be seen in Figure 9, the *CTC Library* follows a modular approach in order to tailor the capabilities to the needs and resources of the device. A support tool for the *CTC Library*, called *CTC Compiler*, is used to process the original schemas and automatically create the *Context Table* and *Template Table*, as well as the necessary native code for the data model bindings (depicted as *Binding Stubs* in Figure 9). This code is embedded in the devices’s application code at programming time and is referenced by the *CTC Library*.

The *Manager* component is responsible for the management of the external accesses (either write or read) to the *Context Table* and *Template Table* including the schema register process.

As explained before, the *Context Table* component holds all of the *Schema Context*s, whereas the *Template Table* component contains the templates of all of the registered schemas.

The *Codifier* component is in charge of decoding/codifying the input data streams based on the *CTC Compression Algorithm* and *Schema Context*s stored in the *Context Table*.

The *Binding Stubs* component groups all of the data model binding code automatically generated by the *CTC compiler*. These code stubs are used to directly map the data stream decoded by the *Codifier* component into native structures and to transform native structures into coded streams through the *Codifier* component. This results in a much more efficient processing of the data models as the parsing of the data model’s original format is completely avoided. On the other hand, the *Generic Serializer* component is used to rebuild the coded data stream to its original format (by processing the data stream and merging the templates) and vice versa.

A resource-constrained device will use the *Binding Stubs* component, that produces native and efficient structures, whereas devices that need access to the original format (such as gateways) will use the *Generic Serializer*. Note that a device that uses the *Binding Stubs* component does not need to include the *Template Table* and *Generic Serializer* components.

## 6. Performance Evaluation

In this section, two performance tests are presented in order to compare CTC and EXI implementations. In the first test, a set of XML instances are compressed by using (a) EXIficient [21], an EXI implementation, and (b) a prototype implementation of the CTC approach. In the second test, the performance of the decoding process and memory usage is analyzed, using as input the compressed streams obtained from the previous test. In order to decode the EXI streams, another EXI implementation more suited to resource-constrained systems is used, Embeddable EXI Processor (EXIP) [22]. EXIP is considered here because, to the authors’ knowledge, it is the best suited to resource-constrained systems. Other implementations targeted to resource-constrained systems, such as the solution WS4D-uEXI [23] promoted by the Web Services for Devices (WS4D) initiative, only implement a subset of the EXI specification and/or are somewhat outdated.

The tests were performed in a CC2650 MCU [24] running at 48 MHz. The test applications, as well as the code under test were compiled with the optimizations turned on.

The set of XML documents used in the tests is composed of the XML Schema instances for the Network Configuration Protocol (*netconf*) [25], Media Types for Sensor Markup Language (*SenML*) [26], Data Types definitions for OPC Unified Architecture of the OPC Foundation (*OPC-UA Types*) [27] and ZigBee Smart Energy Profile 2.0 (*SEP2*) [28], presented in the EXIP evaluation paper [22]. As in [22], three different documents per XML Schema are considered. Additionally, the *Notebook* XML instance used as an example in the EXI Primer web page [19] is also included.

### 6.1. First Comparison: Compression Size

**Figure 10:**
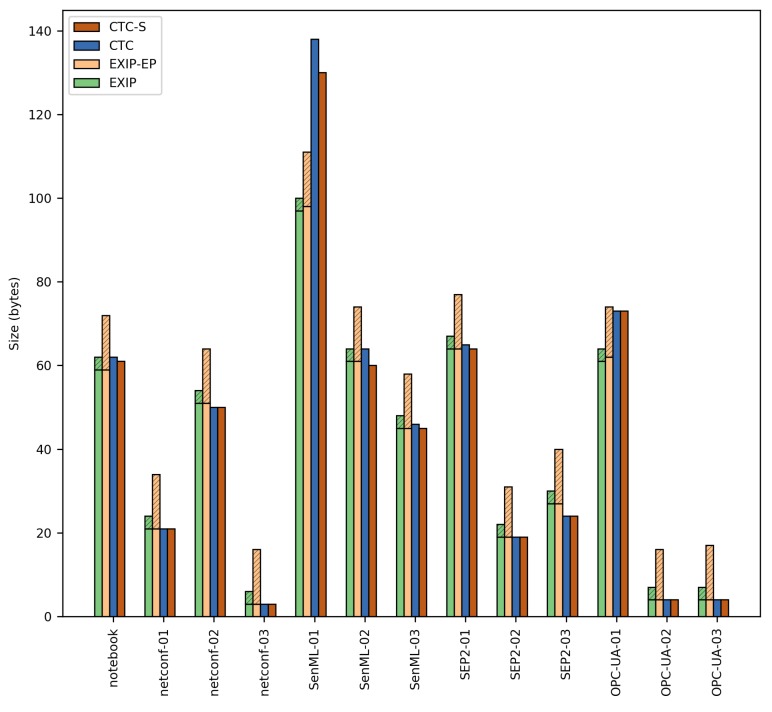
XML document compression comparative in bytes. *EXIP*: schema strict mode, all *preserve* options to False and *schemaId* to constant string “1”. *EXIP-EP*: same options as *EXIP* case plus EXI Profile. *CTC*: CTC normal compression mode. *CTC-S*: CTC strict mode. Stacked columns indicate the extra overhead due to EXI options embedded in the EXI header.

For this comparison, the XML documents were compressed using the EXIficient [21] EXI processor implementation. In order to ensure fairness, the EXI compression options were carefully configured. First, the EXI “schema strict” compression mode was selected. This mode takes into account the XML Schema(s) that describe a XML document in order to achieve the most compact compression. Additionally, all of the EXI *preserve* options were set to False, reducing the overhead that may be produced by compressing meta-data (such as comments). Finally, the EXI *schemaId* option was set to the constant string “1”. This assures that the *schemaId* option is included in the EXI header, but removes the arbitrary overhead of long identifiers. For each XML document, four cases are considered: with/without EXI Profile parameters and with/without including the EXI options in the EXI header. For the CTC case, the *Context Table*s and *Template Table*s were created from the XML Schemas, and the compression was performed using the CTC approach. Results in terms of size are shown in Table 3 and Figure 10.

Results show that CTC has a very similar compression size performance compared to EXI and an average better performance if we take into account the EXI header. The overhead produced by the EXI header can be overcome by providing the EXI options out of band, although this would imply a loss of flexibility. However, it may be necessary in order to reduce communication bandwidth, especially for the EXI Profile case. On the other hand, it is interesting to note that in the case of the *SenML-01* document, EXI shows better compression results. The reason lies in the fact that our proposal is not able to compress the occurrences of strings outside the schema, while EXI does not differentiate between strings belonging to data and schema space.

### 6.2. Second Comparison: Decoding Speed

For the second test, EXI streams produced in the previous compression test were decoded using the EXIP v5.4 [29] EXI implementation. The EXI grammars were statically created from the set of test XML Schemas (using the tools provided by EXIP) and included in the EXIP test code. These EXI grammars were then used at runtime by EXIP to perform the decoding of the EXI streams. In the case of the CTC approach, the *Context Table*s and *Template Table*s were created using the *CTC Compiler* and added to the CTC test code as described in Section 5.2. A prototype implementation of CTC was used to decode the CTC streams produced in the previous test.

We performed 100 runs for each EXI and CTC compressed stream with no additional system load. As in the previous test, we considered four EXI cases for each XML instance document decoding: with/without EXI Profile parameters and with/without including the EXI options in the EXI header. For the EXI Profile cases, EXI Profile options had to be stripped from the EXI header of the EXI compressed streams because they are not supported by EXIP.

**Figure 11:**
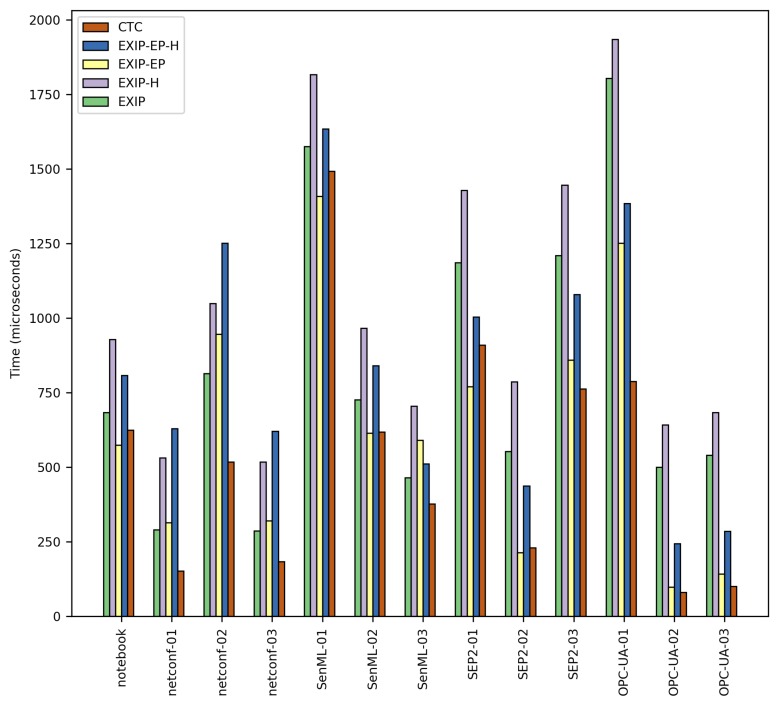
XML document decoding time comparative. *EXIP*: schema strict mode, all *preserve* options to False and *schemaId* to constant string “1”. *EXIP-H*: same options as *EXIP* case and EXI options embedded in the EXI header. *EXIP-EP*: same options as *EXIP* case plus EXI Profile. *EXIP-EP-H*: same options as *EXIP-EP* column and EXI options embedded in the EXI header, but no EXI Profile parameters. *CTC*: CTC normal and strict modes.

Table 4 and Figure 11 show the test results. For the CTC test, only one result column is presented because there is no notable difference between the results yielded by CTC in normal and strict modes.

Results from the tests show that CTC performed generally better in terms of processing time (an average of 36.6% time reduction and up to 87.4% time reduction) compared to any EXIP case. This is a direct result from using the simpler CTC approach and structure of the *Context Table*s and *Template Table*s compared to the EXI specification and EXIP implementation. This difference in decoding speed will be more pronounced in devices with slower CPUs, such as the popular TelosB [30], which runs at 8 MHz. For those cases, times shown in Table 4 will be incremented by a factor of 2–8. Reducing processing time is key in resource-constrained devices in order to reduce the energy consumption as much as possible and to make the most of the available energy.

### 6.3. Third Comparison: Memory Usage

In the last comparison, memory usage of the EXIP library and CTC prototype implementations were compared in terms of required Flash and runtime memories (i.e., code size, data, heap and stack consumption).

**Figure 12:**
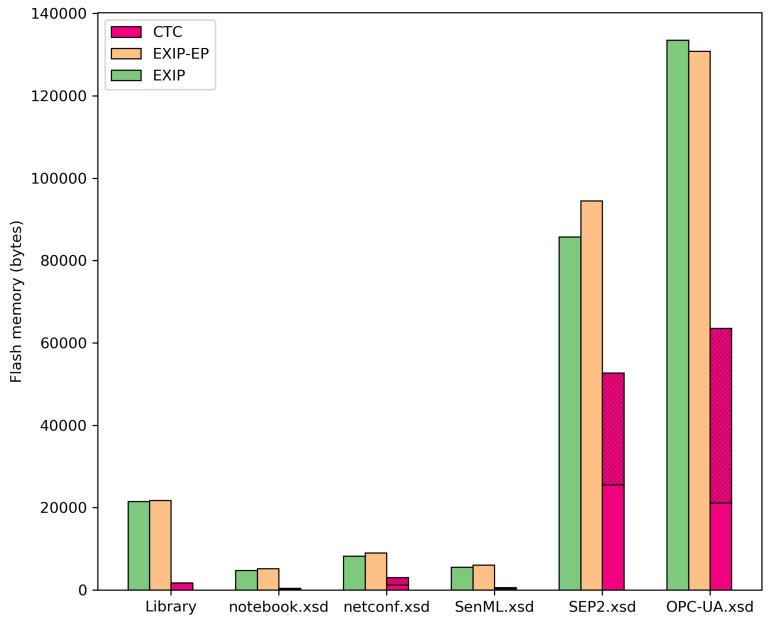
Flash memory consumption comparative in bytes.

The EXIP library supports a dedicated compilation configuration for EXI Profile. In this configuration, the EXIP library code and EXI grammars are more compact, and the RAM usage is notably reduced. Both compilation configurations, normal and EXI Profile, were taken into account in the comparison. For CTC, the memory consumption for the *Context Table*s and *Template Table*s are separately considered. Measures were taken from the test applications used in the second test (described in Section 6.2), and the results are listed in Table 5. Memory usage is separately listed for the EXI and CTC core libraries (labelled as “library”) and for each XML schema (labeled as “*.xsd”). Code memory (Flash) and data memory are depicted in different columns. Figure 12 shows the Flash memory usage listed in Table 5.

Results show that CTC requires significantly less Flash and runtime memory than EXIP. In the case of the base library, CTC is 7.9% the size of the EXIP EXI Profile implementation. For the test XML Schemas, the comparative size ranges from 7.7%–55.8%. As has been explained in Section 5.2, the *Template Table* can be stripped from devices that do not make use of it, thus reducing even further CTC memory requirements for the test XML Schemas. In this case, the comparative memory usage will be reduced to 3.9%–29.0%.

Additionally, CTC uses nearly no data RAM, while EXIP uses 252 bytes in its best case and up to 13,765 bytes in the worst one. Finally, the maximum heap and stack used for the *EXIP* case is 1734 and 904 bytes, respectively, and for the *EXIP-EP* case, heap and stack usage amounts to 1294 and 792 bytes, respectively. In contrast, CTC uses no heap, and the maximum stack size consumed is 692 bytes.

Devices need to share the memory between multiple functionalities (application, sensor drivers, communication stacks, etc.) and will likely need to accommodate more than one schema. Results show that CTC memory requirements are much more suited to resource-constrained devices than EXI implementations due to the significantly smaller memory footprint.

## 7. Conclusions

In this paper we have presented *Context and Template based Compression*, a compression approach for standard data model representation formats. CTC provides a data model representation encoding targeted to resource-constrained devices that is more efficient than standard formats but that allows seamless transformation between the CTC format and the original format. Additionally, CTC supports the WoT paradigm which consists on supporting web standards directly on embedded devices. CTC makes possible the native use of Web Services by enabling the native use of standard data model representation formats Web Services are based on.

Cyber Physical Systems rely on the deployment of interconnected heterogeneous devices and systems. This demands interoperable communications and data models which are typically addressed through the adoption of generic data formats such as XML or JSON. However, the verbosity of these standard data formats requires system resources that might be beyond the capabilities of the resource-constrained devices typically used into CPS. CTC addresses this problem by easing the interoperable integration of heterogeneous devices at the data representation level while requiring very low resource needs (in terms of communication bandwidth, memory size and processing power).

We have shown that CTC provides good performance results compared to EXI implementations. CTC achieves better performance than EXI implementations in terms of speed and memory usage, while keeping a similar efficiency in terms of compression for EXI’s better case (*Schema Strict*). These results show that CTC is a good candidate for resource-constrained devices as it produces very efficient implementations in terms of memory usage and energy consumption.

Additionally, the CTC communication model provides a flexible and interoperable communication architecture. Devices can communicate using standard data model representation formats with devices residing in the same local network or in external networks. The communication can be end-to-end if both devices implement CTC or the gateway can act as an application level gateway otherwise. The schema registration mechanism allows to assign and distribute schema information dynamically at running time. The flexibility in the location of the schema information storing place reduces the message transmission overhead and removes the need to store *Template Tables* on the devices themselves, saving memory resources.

The *CTC Library* provides a modular approach that allows to tailor the capabilities to the needs and resources of the devices. The library is complemented by the *CTC Compiler* tool, easing and automating the implementation of the *Context Table*, *Template Table* and data model bindings to native code.

As a future work, we are considering to extend CTC in order to take into account constraints described in the schema. This would improve the compression and the addition of partial validations to the coded streams. We are also planning on extending the *CTC Compiler* to add support for automatic generation of Web Service bindings.

Another line of research is to apply the concepts used in CTC to EXI grammars representation. By improving the implementation efficiency of EXI processors (from in-memory representation to grammar processing), the use of EXI will open to a wider range of resource-constrained devices.

## Figures and Tables

**Table 1:** *Schema Context* table example. Each column represents an *eContext*. The content of the *Children* row represents the tuple (*Template*, *Type*, *IsOptional*, *IsArray*, *Context*). *x* denotes *complex*, *s string*, *f* False and *t* True.

Attribute	Id		
	ROOT (0)	C1 (1)	C2 (2)
MultipleParents	f	f	t
Order	fixed	dynamic	fixed
Children	e1(t1,x,f,t,C1) e2(t2,x,t,f,C2)	e3(t3,s,f,f,-) e4(t4,s,t,f,-) e5(t5,x,f,t,C2)	e6(t4,s,t,f,-)

**Table 2:** *Schema Context Notebook* example, after *Context Collapsing*. The content of the *Children* row represents the tuple (*Template*, *Type*, *IsOptional*, *IsArray*, *Context*). C*n* represents the *eContext* *Id* and t*n* the template identifier; *x* denotes the *complex* value, *s* *string*, *c* the *constant*, *d* the *date-time*, *t* True and *f* False.

Attribute	Id				
	C1 (ROOT)	C2 (CONTENT)	C3 (NOTEBOOK)	C4 (NOTE)	C5 (NOTE-ATT)
MultipleParents	f	f	f	f	f
Order	fixed	choice	fixed	fixed	dynamic
Children	(t1,c,t,f,-) (-,x,f,f,C2)	(t2,x,t,f,C3) (t3,d,t,f,-)	(t3,d,t,f,-) (t4,x,f,t,C4)	(-,x,f,f,C5) (-,s,f,f,-) (-,s,f,f,-)	(t3,d,f,f,-) (t5,s,t,f,-)

**Table 3:** XML document compression comparative in bytes. *EXIP*: schema strict mode, all *preserve* options to False and *schemaId* to constant string “1”. *EXIP-EP*: same options as *EXIP* column plus EXI Profile. *CTC*: CTC normal compression mode. *CTC-S*: CTC strict mode. Numbers inside brackets indicate the extra overhead due to EXI options embedded in the EXI header. The list of XML documents stand for: *Notebook*, EXI *Notebook* example; *netconf*, Network Configuration Protocol; *SenML*, Sensor Markup Language; *SEP2*, ZigBee Smart Energy Profile 2.0; *OPC-UA*, OPC Unified Architecture.

XML	Original	EXIP	EXIP-EP	CTC	CTC-S
*Notebook*	297	(3+) 59	(13+) 59	62	61
*netconf-01*	395	(3+) 21	(13+) 21	21	21
*netconf-02*	660	(3+) 51	(13+) 51	50	50
*netconf-03*	423	(3+) 3	(13+) 3	3	3
*SenML-01*	448	(3+) 97	(13+) 98	138	130
*SenML-02*	219	(3+) 61	(13+) 61	64	60
*SenML-03*	173	(3+) 45	(13+) 45	46	45
*SEP2-01*	406	(3+) 64	(13+) 64	65	64
*SEP2-02*	92	(3+) 19	(12+) 19	19	19
*SEP2-03*	522	(3+) 27	(13+) 27	24	24
*OPC-UA-01*	936	(3+) 61	(12+) 62	73	73
*OPC-UA-02*	278	(3+) 4	(12+) 4	4	4
*OPC-UA-03*	300	(3+) 4	(13+) 4	4	4

**Table 4:** XML document decoding time comparative. Numbers are in microseconds. *EXIP*: schema strict mode, all *preserve* options to False and *schemaId* to constant string “1”. *EXIP-H*: same options as *EXIP* column and EXI options embedded in the EXI header. *EXIP-EP*: same options as *EXIP* column plus EXI Profile. *EXIP-EP-H*: same options as *EXIP-EP* column and EXI options embedded in the EXI header, but no EXI Profile parameters. *CTC*: CTC normal and strict modes. The list of XML documents stand for: *Notebook*, EXI *Notebook* example; *netconf*, Network Configuration Protocol; *SenML*, Sensor Markup Language; *SEP2*, ZigBee Smart Energy Profile 2.0; *OPC-UA*, OPC Unified Architecture.

XML	EXIP	EXIP-H	EXIP-EP	EXIP-EP-H	CTC
*Notebook*	684	929	574	808	625
*netconf-01*	290	531	314	629	152
*netconf-02*	814	1049	946	1251	518
*netconf-03*	286	518	320	621	183
*SenML-01*	1576	1817	1409	1635	1493
*SenML-02*	726	966	615	840	618
*SenML-03*	465	705	591	511	377
*SEP2-01*	1186	1429	770	1004	910
*SEP2-02*	553	787	213	437	230
*SEP2-03*	1210	1446	860	1079	763
*OPC-UA-01*	1804	1935	1251	1385	788
*OPC-UA-02*	500	642	98	244	81
*OPC-UA-03*	540	684	142	285	101

**Table 5:** Memory consumption comparative in bytes. The table shows the use of code memory (Flash) and data memory (RAM). Additionally, the maximum heap and stack used for the EXIP case is 1734 and 904 bytes respectively. For the EXIP-EP case, maximum heap and stack usage amounts to 1294 and 792 bytes respectively. Finally, CTC uses no Heap and the maximum stack size used for the tests is 692 bytes.

XML Schema	Flash			Data RAM		
	EXIP	EXIP-EP	CTC	EXIP	EXIP-EP	CTC
Library	21,493	21,794	1722	292	292	12
notebook.xsd	4745	5242	208 + 196	1196	252	0
netconf.xsd	8226	8979	1224 + 1812	1748	292	0
SenML.xsd	5550	6064	320 + 300	1348	292	0
SEP2.xsd	85,776	94,500	25,560 + 27,188	11,860	292	0
OPC-UA.xsd	133,528	130,823	21,172 + 42,396	13,765	292	0
